# Comparative genomics of the syndecans defines an ancestral genomic context associated with matrilins in vertebrates

**DOI:** 10.1186/1471-2164-7-83

**Published:** 2006-04-18

**Authors:** Ritu Chakravarti, Josephine C Adams

**Affiliations:** 1 Dept. of Cell Biology, Lerner Research Institute, Cleveland Clinic Foundation, Cleveland, OH 44195, USA; 2 Dept. of Molecular Medicine, Cleveland Clinic Lerner College of Medicine of Case Western Reserve University, Cleveland Clinic Foundation, Cleveland, OH 44195, USA

## Abstract

**Background:**

The syndecans are the major family of transmembrane proteoglycans in animals and are known for multiple roles in cell interactions and growth factor signalling during development, inflammatory response, wound-repair and tumorigenesis. Although syndecans have been cloned from several invertebrate and vertebrate species, the extent of conservation of the family across the animal kingdom is unknown and there are gaps in our knowledge of chordate syndecans. Here, we develop a new level of knowledge for the whole syndecan family, by combining molecular phylogeny of syndecan protein sequences with analysis of the genomic contexts of syndecan genes in multiple vertebrate organisms.

**Results:**

We identified syndecan-encoding sequences in representative Cnidaria and throughout the Bilateria. The C1 and C2 regions of the cytoplasmic domain are highly conserved throughout the animal kingdom. We identified in the variable region a universally-conserved leucine residue and a tyrosine residue that is conserved throughout the Bilateria. Of all the genomes examined, only tetrapod and fish genomes encode multiple syndecans. No syndecan-1 was identified in fish. The genomic context of each vertebrate syndecan gene is syntenic between human, mouse and chicken, and this conservation clearly extends to syndecan-2 and -3 in *T. nigroviridis*. In addition, tetrapod syndecans were found to be encoded from paralogous chromosomal regions that also contain the four members of the matrilin family. Whereas the matrilin-3 and syndecan-1 genes are adjacent in tetrapods, this chromosomal region appears to have undergone extensive lineage-specific rearrangements in fish.

**Conclusion:**

Throughout the animal kingdom, syndecan extracellular domains have undergone rapid change and elements of the cytoplasmic domains have been very conserved. The four syndecan genes of vertebrates are syntenic across tetrapods, and synteny of the syndecan-2 and -3 genes is apparent between tetrapods and fish. In vertebrates, each of the four family members are encoded from paralogous genomic regions in which members of the matrilin family are also syntenic between tetrapods and fish. This genomic organization appears to have been set up after the divergence of urochordates (*Ciona*) and vertebrates. The syndecan-1 gene appears to have been lost relatively early in the fish lineage. These conclusions provide the basis for a new model of syndecan evolution in vertebrates and a new perspective for analyzing the roles of syndecans in cells and whole organisms.

## Background

Proteoglycans are important and ancient mediators of cell interactions in metazoan organisms. In the simplest multicellular animals, sponges, extracellular proteoglycans contribute to adhesion between cells and processes of self-recognition and host defence [1,2]. The syndecans are the only major family of transmembrane proteoglycans and are conserved in metazoa from nematode worms to man. The genomes of invertebrates such as *C. elegans *and *D. melanogaster *contain a single syndecan gene, whereas mammalian genomes contain four syndecan genes [3,4]. In all the organisms in which they have been studied, syndecans have important roles in cell interactions, adhesion, migration and receptor signaling. The single syndecans of *C. elegans *and *D. melanogaster *are required for proper axon guidance during development of the nervous system [5-8]. *C. elegans *syndecan also functions in vulval development [9]. The vertebrate syndecans each have distinct tissue expression patterns and distinct functional attributes. For example, syndecan-1 is expressed in epithelia and certain mesenchymal cells. Syndecan-1 null mice show deficits in cell migration behaviors associated with inflammatory response and wound-healing [10,11]. In single cells, syndecan-1 activates formation of filopodia and lamellipodia that mediate cell motility [12,13]. Syndecan-2 is expressed by mesenchymal, liver and neuronal cells. It participates in the development of the left/right body axis during *Xenopus *development [14], has been identified to function in the assembly of neuronal dendritic spines and in ECM assembly in mammalian systems, and is essential for angiogenesis in zebrafish [15-17]. Syndecan-3 is mostly expressed in the nervous system and is important in hippocampal function, particularly in controlling feeding behavior and hippocampus-dependent memory [18,19]. Syndecan-4 is broadly expressed by many tissues and cell types and has specific signaling roles as an integrin co-receptor in the assembly of focal adhesions. It also contributes to angiogenesis and wound-healing in mice [4,20,21].

In mammals, syndecans have significant clinical relevance in the pathologies of infection, cancer and wound repair. Syndecan-1 is a receptor for human immunodeficiency virus in cell culture [22] and contributes to the pathology of infection by *Pseudomonas aeruginosa *and *Staphylococcus aureus *[23-25]. Syndecan-1 deficient mice have increased resistance to wingless-1-mediated tumorigenesis [26]. Altered expression of syndecan-1 has been documented in many human carcinomas, lymphomas and in multiple myeloma; in multiple myeloma syndecan-1 has been proposed for clinical use as a prognostic marker [27-34]. Other studies have implicated syndecans in tissue repair. Thus, the lack of syndecan-1 or -4 in mice results in impaired inflammatory responses, wound-healing, and angiogenesis [10,21,35]. Studies in animal models have demonstrated increased expression of syndecan-1, -3 and -4 after cardiac injury, indicating possible roles in cardiac remodeling [36-38].

In structure, all syndecans consist of an extracellular domain with sites for attachment of glycosaminoglycan (GAG) sidechains, a single transmembrane domain and a short cytoplasmic domain. Through their GAG chains, syndecans act as co-receptors for the cell-surface binding of heparin-binding growth factors such as bFGFs (basis fibroblast growth factor) and Wnt (wingless) [3,4,26,39]. The activity of invertebrate syndecans in axon guidance depends on co-receptor activity in the slit/Robo pathway [5,8]. Direct protein-protein interactions of the extracellular domain have also been implied for syndecan-1 and -4 [40]. Based on comparisons of mammalian, *C. elegans *and *D. melanogaster *syndecans, the cytoplasmic domains are recognized to contain two conserved regions, designated C1 and C2, that are present in all these syndecans, and a central variable, (V), region that is unique to each form of syndecan. The V regions themselves are very highly conserved between species orthologues of syndecans -1 to -4, suggestive of important specific functions [3,4]. In general, the cytoplasmic domains participate in the assembly of juxtamembrane complexes that regulate cell signaling and the organizational state of the actin cytoskeleton. The C2 region binds PDZ-containing proteins that form multiprotein scaffolds by oligomerization and which also mediate syndecan recycling [41]. Cask, src tyrosine kinase and synectin are known binding partners of the C1 region. The V regions have been most intensively studied in syndecan-2 and syndecan-4, and have specific binding partners that contribute to cell signaling that regulates cytoskeletal structures [3,4,15]. On the basis of their protein sequences, the four mammalian syndecans group into two pairs: syndecan-1 and -3 have higher sequence identity with each other, as do syndecan-2 and syndecan-4 [3,4].

To date, the relationships of invertebrate and vertebrate syndecans have been considered only at the level of their protein sequences. With regard to these analyzes, there are gaps in our knowledge of chordate syndecans, as the most intensive studies have focused on mammalian and amphibian syndecans [e.g., [3,4,10,11,14,18,20,21]]. Invertebrate syndecans have been experimentally studied in *D. melanogaster *and *C. elegans*, but little is known about other invertebrate syndecans. It is now recognized that the genomes of *D. melanogaster *and *C. elegans *have evolved rapidly with extensive gene loss [42,43]. Thus, one purpose of our study was to capitalize on the recent sequencing of the genomes of the basal chordate, *Ciona intestinalis*, the chicken and three species of fish, along with the expanded knowledge from the genomes of the sea urchin *Stronglyocentrotus purpuratus*, the amphioxus *Branchiostoma floridae*, multiple representatives of the Cnidaria, and the many expressed sequence tag projects directed to invertebrate species, to obtain a comprehensive view of this important proteoglycan family at protein sequence level. A second purpose has been to develop a new level of knowledge for the whole syndecan family, by analyzing the genomic contexts of syndecan genes in multiple organisms. The comparative genomics of gene families is a powerful approach to understanding the relationships between the members of multigene families, either within a single genome or through comparison of the conservation of genomic context across the genomes of multiple organisms. In addition, ideas about the origins of gene families can be clarified through the study of the genomes of modern animals from different phyla. The insights obtained from comparative genomics are relevant to making choices of model organisms for experimental purposes and may advance understanding of the roles of gene families at systems level [44].

## Results

### Molecular phylogeny of syndecans across the animal kingdom

Our searches of recently completed genome sequences and the database of expressed sequence tags produced an expanded dataset of syndecans that demonstrates remarkable conservation of this proteoglycan family across the animal kingdom. Syndecans were identified in multiple species of Cnidaria as well as throughout the Bilateria. The genomes of *Ciona intestinalis*, *Stronglyocentrotus purpuratus *and amphioxus (*Branchiostoma floridae*) encode single syndecans and multiple syndecans are encoded in fish and other vertebrate genomes (Table 1). However, no syndecan-1 sequence was identified in fish, nonwithstanding extensive searches of three fish genomes and the large database of expressed sequence tags from many teleost fish species.

The sequence relationships of selected invertebrate and fish syndecans to human syndecans were examined by calculating WU-BLASTP conservation scores [45]. *X. laevis *syndecans were included for comparison. Whereas each of the *X. laevis *and *T. nigroviridis *syndecans was clearly most closely related to one of the human syndecans (e.g., *T. nigroviridis *syndecan-3 has the highest score with human syndecan-3, etc), each of the invertebrate syndecans had similar low conservation scores with all the human syndecans and in some cases no hit was obtained (Table 2). This suggests that the sequences of invertebrate syndecans are similarly related to all four of the vertebrate syndecans.

**Figure 1:**
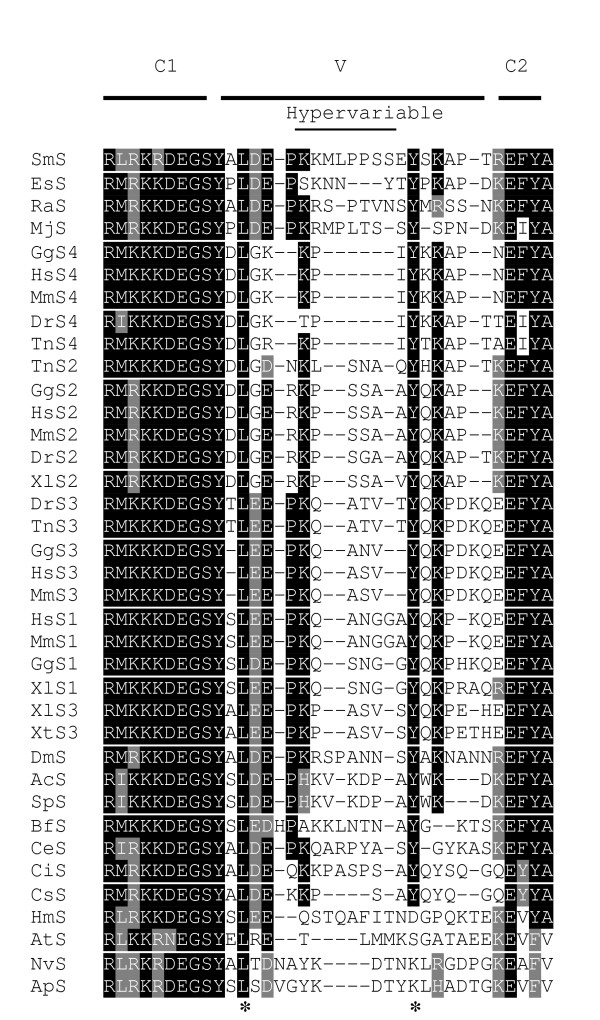
**Conservation of syndecan cytoplasmic domains**. Amino acid sequences of syndecan cytoplasmic domains from different species were aligned in ClustalW. The alignment is presented in Boxshade 3.2. Black shading shows identical amino acids; gray indicates conservative substitutions and no shading indicates unrelated amino acids. V = variable region and the very highly-conserved leucine and tyrosine residues are indicated by asterisks. The central hypervariable region (13) is also indicated. Key: Ac, *Anthocidaris crassispina*; Ap, *Acropora palmata*; At, *Acropora tenuis*; Bf, *Branchiostoma floridae*; Ce, *Caenorhabditis elegans*; Ci, *Ciona intestinalis*; Cs, *Ciona savignyi*; Dm, *Drosophila melanogaster*; Dr, *Danio rerio*; Es, *Euprymna scolopes*; Gg, *Gallus gallus*; Hm, *Hydra magnipapillata*; Hs, *Homo sapiens*; Mj, *Marsupenaeus japonicus*; Mm, *Mus musculus*; Nv, *Nematostella vectensis*; Rp, *Rhipicephalus appendiculatus*; Sm, *Schmidtea mediterranea*; Sp, *Strongylocentrotus purpuratus*; Tn, *Tetraodon nigroviridis*; Xl, *Xenopus laevis*; Xt,*Xenopus tropicalis*. All syndecans are abbreviated as S.

The cytoplasmic domains have been recognized as the most conserved region of syndecan protein sequences [3,4]. To examine the conservation of cytoplasmic domains across the new dataset, we aligned the sequences by the CLUSTALW progressive local alignment method [46]. The alignment demonstrated near complete conservation of the C1 region in all syndecans, with some variation at the second and third positions in the vertebrate syndecan-2 group, *D. rerio *syndecan-4, and many of the invertebrate syndecans (Figure 1). The C2 region is almost universally conserved in Bilateria as EFYA, with the exception of *Ciona *syndecans that contain EYYA and fish syndecan-4s that contain EIYA. Greater variability of the C2 motif is evident in the Cnidaria (Figure 1). All of these variants are predicted to be functional PDZ-binding motifs [47].

The alignment also demonstrated that the invertebrate syndecans from different phyla contain distinctive V regions, all of which are different from those of syndecan-1, -2, -3 and -4. In vertebrate syndecans, the syndecan-4 V region is well-conserved between fish and tetrapods. In the syndecan-2 group, the V region of *D. rerio *syndecan-2 is very similar to that of tetrapod syndecan-2s, whereas in *T. nigroviridis *the central hypervariable region is distinct at 3 residues. The V regions of syndecan-3s are well-conserved between fish, chicken, mouse and human, yet the V region of the *Xenopus *syndecan-3s have unique features that result in a separate clustering in the sequence alignment (Figure 1). Most strikingly, the alignment highlighted the universal conservation of the first leucine residue in the V regions of all animals. A single tyrosine residue is also very well-conserved across the Bilateria but is not present in any of the V regions from the Cnidaria (asterisks in Figure 1).

**Figure 2:**
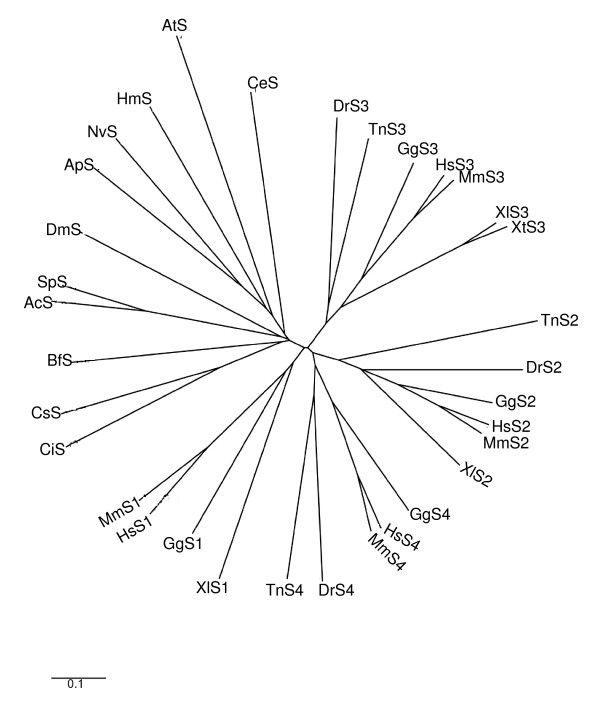
**Sequence relationships of invertebrate and vertebrate syndecans**. The amino acid sequences of all the full-length syndecans in the dataset were aligned in TCOFFEE. The Phylip distance matrix output was used to prepared an unrooted phylogenetic diagram in DRAWTREE. Key is the same as in Figure 1. Scale bar = 0.1 substitution/site.

To compare all the full-length sequences in the dataset, we used the TCOFFEE alignment algorithm that combines pairwise/global and local alignment methods into a single model and is more accurate than CLUSTALW for sequences of lower identity [48]. The Phylip distance matrix output was used to prepare an unrooted tree. This analysis confirmed the absence of syndecan-1 from the fish species. Fish and amphibian syndecan-2, -3 and -4 all grouped clearly with their tetrapod counterparts. Each set of syndecan-1, -2, 3- and -4 sequences formed a distinct group within the phylogenetic diagram, and the syndecan-2 and -4 groups appeared more closely related to each other than to other syndecans. *X. laevis *syndecan-1 has unusual histidine-rich sequence features [49] and therefore forms an deep-branching node with tetrapod syndecan-1s; nevertheless the syndecan-1 grouping was clear (Figure 2; the sequence of *X. tropicalis *syndecan-1 is similar to that of *X. laevis*; *X. tropicalis *syndecan-1 is present on scaffold 100 in genome assembly 4.1) [50].

**Figure 3:**
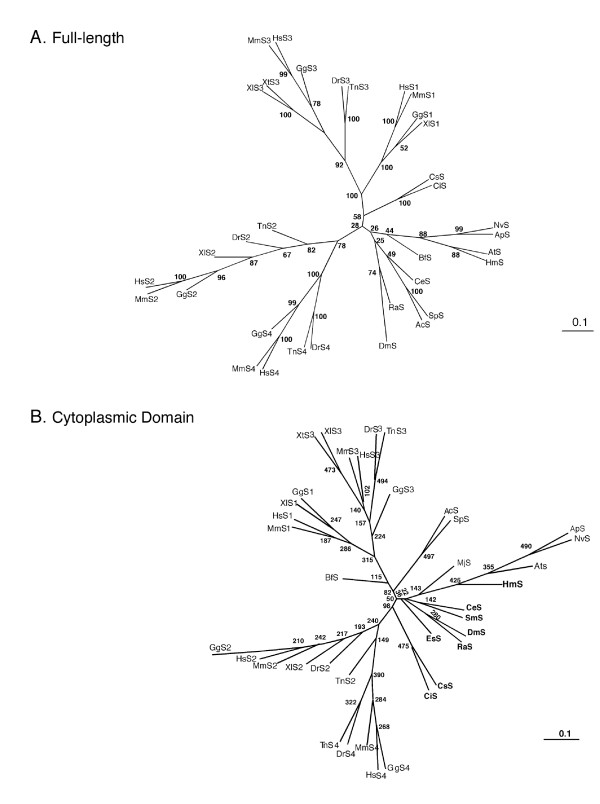
**Phylogenetic trees of full-length and cytoplasmic domain of syndecans**. The CLUSTALW outputs of TCOFFEE alignments of the amino acid sequences of (A), full-length, and (B), cytoplasmic domains, of syndecans in our dataset were used to generate unrooted phylogenetic trees by the maximum likelihood reconstruction method, PHYML, using the WAG substitution model. Bootstrap analysis was run for 100, (A), or 500, (B), cycles and the bootstrap replication values are shown at each node. Scale bars = 0.1 substitution/site.

To evaluate the robustness of these groupings, further analyses of the full-length sequences and the cytoplasmic domains were conducted using the program PHYML, that estimates phylogenies based on the maximum-likelihood principle [51], with inclusion of bootstrap analysis [52]. The unrooted tree derived from the full-length sequences confirmed the distinct groupings of the sets of syndecan-1, -2, -3 and -4 and also demonstrated the syndecan-1/syndecan-3 and syndecan-2/syndecan-4 pairings. These topologies were supported by robust bootstrap values (at least 67%, with the exception of the *G. gallus */*X. laevis *syndecan-1 node) (Figure 3A). However, the positioning of invertebrate syndecans relative to vertebrate syndecans was not well supported, likely due to the extent of divergence of their extracellular domain sequences (Figure 3A). Most of the subgroupings within the invertebrates appeared biologically meaningful and were well-supported by the bootstrap analysis, with the exception of the placements of the amphioxus sequence at a node with Cnidarian syndecans and *C. elegans *at a node with echinoderm syndecans (Figure 3A). It is likely that these anomalies are an artifact of long branch attraction. At present, there are no additional cephalochordate or nematode syndecan sequences to include in the dataset. The same general topologies for the vertebrate syndecans were supported in the tree prepared from the cytoplasmic domains. Again, not all nodes within the invertebrate syndecan group were biologically meaningful -*M. japonicus *syndecan was placed as a outgroup of the Cnidaria – and the central nodes between vertebrate and invertebrate syndecans were not robust (Figure 3B). In summary, the multiple molecular phylogenetic methods of analysis of syndecan protein sequences support the pairings of syndecan-1 with syndecan-3, and syndecan-2 with syndecan-4, and indicate that the invertebrate syndecans are distantly and equivalently related to the four vertebrate syndecans.

### Vertebrate syndecan genes show extensive conservation of synteny

**Figure 4:**
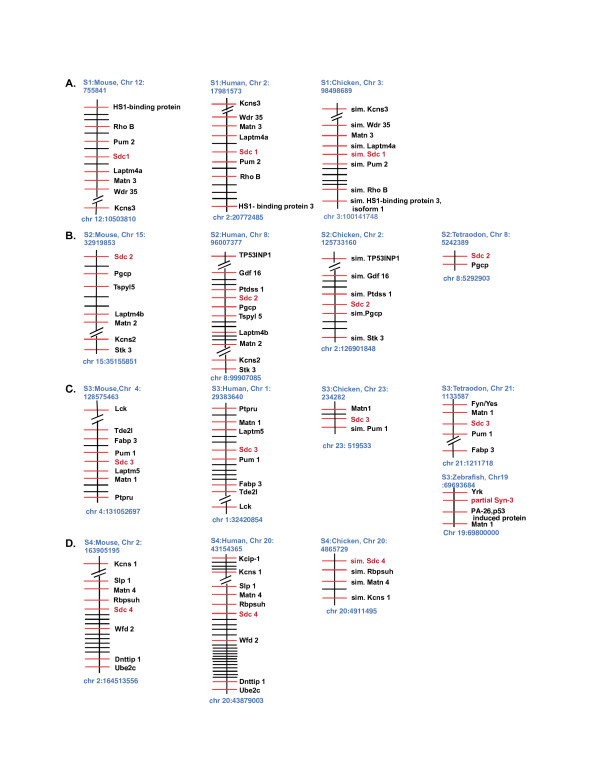
**Conservation of synteny between vertebrate syndecan genes**. The genomic contexts of: (A), syndecan-1 genes; (B), syndecan-2 genes; (C), syndecan-3 genes and (D), syndecan-4 genes, were analysed for conservation of neighboring genes in the human, mouse, chicken, *T. nigroviridis *and *D. rerio *genomes. Each diagram represents the order of genes along a portion of the indicated chromosomes. Each horizontal line represents a gene: red lines represent genes that are syntenic and black lines represent non-conserved intervening genes. The numbers indicate the exact location in nucleotides of each region on its respective chromosome in each species. HUGO gene names are given, and the syndecan genes are indicated in red.

To obtain a clearer perspective on the relationships of the four vertebrate syndecans, we evaluated the vertebrate syndecan gene family at genomic level by comparing the conservation of the gene neighbors of each syndecan gene between the mapped genomes of human, mouse, chicken and, where available, fish. In each genome, each syndecan gene is located on a different chromosome. For each family member, a set of conserved gene neighbors could be identified. For the syndecan-1 gene, six local neighboring genes (*HS1BP, RHOB, PUM2, LAPTM4A*, *MATN3 *and *WDR35*) and, more remotely, *KCNS3*, were conserved in all three species (Figure 4A).

For the syndecan-2 gene, *STK3 *and *PGCP *were conserved as neighbouring genes in human, chicken and mouse. *TSPYL5, LAPTM4B, MATN2 *and *KCNS2 *genes were also conserved between human and mouse and *PTDSS1, GDF16 *and *TP53INP *were also conserved between human and chicken (Figure 4B). In comparing the locus of the syndecan-2 gene in *T. nigroviridis, PGCP *was also conserved as a neighboring gene (Figure 4B). *MATN2 *was not present in this region, but additional searches identified that it is indeed encoded on chromosome 8 (discussed further below). In *D. rerio*, the syndecan-2 gene on chromosome 5 has no gene neighbors in common with those of tetrapods or *T. nigroviridis*, and we infer that this gene underwent a lineage-specific transposition (data not shown).

*PUM1 *and *MATN1 *were conserved gene neighbors of the syndecan-3 gene in human, mouse, chicken and *T. nigroviridis*, and *MATN1 *also mapped adjacent to *D. rerio SDC3 *(Figure 4C). The most extensive local synteny was between human and mouse: *TDE2l, FABP3, LAPTM5 *and *PTPRU *were all conserved as local gene neighbors (Figure 4C). Of these genes, *FABP3 *was also identified to be syntenic with *SDC3 *in *T. nigroviridis*. The gene encoding the tyrosine kinase Lck was also local to *SDC3 *in human and mouse. In the two fish genomes, genes encoding tyrosine kinases were also adjacent to *SDC3*, however the gene products were most similar to other members of the tyrosine kinase family (Figure 4C).

For the syndecan-4 gene, strong conservation of the genomic regions was apparent between human, mouse and chicken: *KCNS1, MATN4 *and *RBPSUHL *were conserved between all three species and additional local genes were conserved between human and mouse (Figure 4D). *SDC4 *is not yet mapped to a chromosome in either the *T*. *nigroviridis *or *D. rerio *genomes.

The high conservation of the genomic context of vertebrate syndecan genes prompted us to examine whether this synteny extends to the urochordate, *Ciona intestinalis*. The urochordate group are basal in the chordate lineage and have not undergone whole genome duplications [53]. The single syndecan gene of *C. intestinalis *is encoded on chromosome 2, scaffold 125 [54]. The *C. intestinalis *genome encodes a single matrilin (Gene Cluster 14563), however, this is located on scaffold 259 on chromosome 1. (This gene product has highest identity to matrilin-3. The Gene Cluster 00035/06628 that is assigned as "matrilin-2" at the Ghost website has highest identity to fibrillin). Of the other gene families consistently represented on the same chromosomes as tetrapod syndecan genes, only *Gdf16 *was identified on *C. intestinalis *chromosome 2 (scaffolds 213 and 58). Ten local gene neighbours of the syndecan gene of *C. intestinalis *are not syntenic with any human or chicken syndecan gene (data not shown). Thus, there are many differences in the genomic context of *C. intestinalis *syndecan.

### Paralogous locations of syndecan and matrilin genes in the human genome

Overall, the data on the genomic contexts of vertebrate syndecan genes demonstrated two striking points. First, in considering the individual family members, there is clear conservation of synteny between fish and tetrapods for each of the syndecan-2, -3 and -4 genes. This indicates that these loci must each have been present in the last common ancestor of fish and tetrapods. Secondly, a deeper level of homology is apparent, in that the syndecan-1, -2, -3 and -4 genes in the organisms we examined all have conserved gene neighbours that are paralogous members of the same gene families. The clearest example of this are the matrilins, that are components of extracellular matrix and which in tetrapods also comprise a gene family of four members [55,56]. With the exception of chicken syndecan-2, a matrilin gene is located near to every examined tetrapod syndecan gene. The same trend was apparent for the fish syndecan-3 genes (Figure 4). Members of the *LAPTM4 *and *KCNS *gene families were also present in the local genomic region of most of the tetrapod syndecan genes. The two members of the pumilo gene family were conserved adjacent to the syndecan-1 and -3 genes (Figure 4).

**Figure 5:**
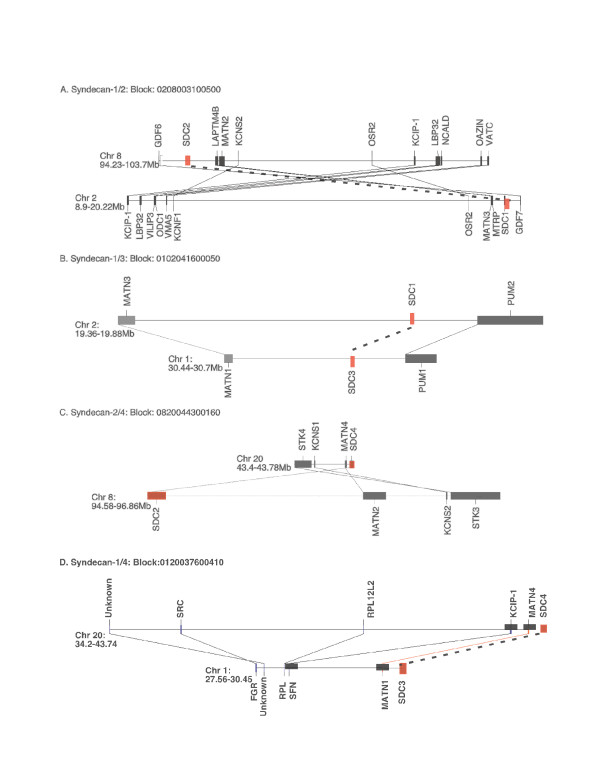
**Paralogous locations of syndecan genes within the human genome**. Paralogous regions covering all four members of the syndecan gene family were identified in the human genome, from the "dataset of paralogons in the human genome v5.28" 57. Each block number and the exact location of the paralogous regions on the respective chromosomes are also shown. Shaded lines represent how the original blocks were modified to include missing syndecan genes (see methods).

These findings strongly suggest that, in each genome, the four syndecan genes are located in paralogous genomic regions that have been conserved throughout the evolution of vertebrates. The existence of such regions in vertebrate genomes provides evidence of whole genome duplication events that took place early in chordate evolution [57-60]. In the lineage of each of the organisms examined, subsequent gene loss or localized rearrangements have blurred the initial four-fold replication of the region in distinct ways over time [61]. In the human genome, the rate of DNA rearrangement is slower than in rodents [61] and paralogous regions have been identified globally by BLASTP-based searches of the genome against itself [57,58]. To substantiate the view obtained from our analysis of local genes on an individual basis, we examined whether the human genome contains evidence of chromosomal paralogies in the regions of the four syndecan genes according to this unbiased independent method. Searches were made through the database of paralogons in the human genome, v5.28 [57]. Strikingly, the genomic region of each human syndecan gene was found to be related to the genomic region of all the other family members. Members of the matrilin family were included in all blocks, and other gene families identified in our local searches were represented in individual paired blocks along with genes that are more distant on each chromosome (Figure 5). Together, these findings provide strong evidence that the four vertebrate syndecan genes have evolved as a consequence of rounds of duplication of a single ancestral chromosomal region.

### The absence of syndecan-1 in fish: analysis of the genomic contexts of matrilin genes in T. *nigroviridis *and *D. rerio*

**Figure 6:**
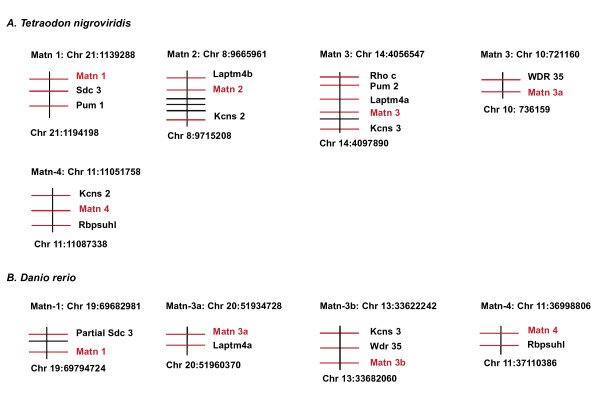
**Genomic contexts of matrilin genes in *T. nigroviridis *and *D. rerio***. The genomic contexts of matrilin gene family members from (A), *T. nigroviridis *and (B), *D. rerio*, were analysed for conservation of neighboring genes with human, mouse and chicken, as shown in Fig. 3, and with each other. Partial synteny was identified. Each diagram represents the order of genes along a portion of the indicated chromosomes. Each horizontal line represents a gene: red lines represent genes that are syntenic and black lines represent non-conserved intervening genes. The numbers indicate the exact location in nucleotides of each region on its respective chromosome in each species. HUGO gene names are given, and the matrilin genes are indicated in red.

Because synteny of syndecan-2 and -3 genes is conserved between tetrapods and pufferfish, and because all four syndecan gene loci show evidence of paralogy in tetrapods, we inferred that the absence of syndecan-1 from fish is a derived characteristic. This raised the question of whether other parts of the conserved chromosomal region are present in fish, even though the syndecan-1 gene itself is missing. We addressed this question through the proximity of syndecan and matrilin genes. In all tetrapods examined, *MATN3 *is close to *SDC1 *(Figure 4). All four forms of matrilin are present in *T. nigroviridis *(Table 3) and both *T. nigroviridis *and *D. rerio *matrilins include two matrilin-3 paralogues (Table 3) [62]. The existence of such paralogous pairs is consistent with the strong evidence for an additional whole genome duplication early in the evolution of teleost fish [63]. We examined the genomic contexts of matrilin genes in *T. nigroviridis *and *D. rerio*. In *T. nigroviridis*, all four matrilins, including *MATN3*, are encoded within genomic regions syntenic to those of tetrapod matrilins (Figure 6A). *MATN2 *is encoded at a distinct point on chromosome 8 from *SDC2*, yet the adjacent genes include *LAPTM4B *and *KCNS2*, that are conserved neighbours in tetrapods. Similarly, *KCNS2 *is adjacent to *MATN4 *(Figure 6A). We hypothesized that fish matrilin-3s should be encoded within a genomic region similar to that of tetrapod matrilin-3 and syndecan-1. Importantly, the order of genes adjacent to *T. nigroviridis MATN3 *matched closely with those adjacent to tetrapod *MATN3*: however, in *T. nigroviridis*, *PUM2 *and *LAPTM4A *are adjacent to each other, suggesting a specific loss of *SDC1*. Although *WDR35 *is not present on chromosome 14, we found that this gene is located on chromosome 10 adjacent to the *T. nigroviridis *paralogue of *MATN3a *(Figure 6A).

The genomic contexts of *D. rerio *matrilin genes, (some of which were previously mapped by the radiation hybrid method [62] and all of which are now physically mapped in Zebrafish genome assembly Zv5), demonstrated the same points, although the conservation of local neighboring genes was not as extensive for *MATN1 *as in *T. nigroviridis *(Figure 6B). In the case of *MATN4*, the local neighboring genes did not match those of tetrapod *MATN4*, but *RBPSUHL *is conserved as a neighboring gene between *D. rerio *and *T. nigroviridis *(Figure 6B). As in *T. nigroviridis, MATN3a *and *MATN3b *both have gene neighbors that match those of tetrapod *MATN3*. However, the neighbors of each *MATN3 *paralogue represent the opposite sides of the conserved genomic region of tetrapods, indicating a break of the chromosome after the additional genome duplication [62]. We did not identify *PUM2 *in the *D. rerio *genome (assembly Zv5). We examined the genomic regions of fish matrilin-3s, as shown in Figure 6, more closely for evidence of syndecan-1 coding sequence. BLAT search of the *Tetraodon *genome with the mRNA nucleotide sequences of human, chicken, or *X. laevis *syndecan-1 did not yield significant findings: in each case, only short, 10–20 nucleotide regions on many different chromosomes were identified as homologous. Syndecan-1 protein sequence searches identified only the cytoplasmic domain of syndecan-2 on chromosome 21. Pairwise BLAST searches of syndecan-1 coding sequences against the *Tetraodon *or *Danio *genomic sequence regions did not identify significant matches. Thus, the syndecan-1 coding sequence is not represented in this genome. In conclusion, our findings provide further evidence for partial synteny of the genomic contexts of syndecan and matrilin genes between fish and tetrapods. Most importantly, the fish genomes provide evidence of complex genomic rearrangements in the region of the matrilin-3 gene that have involved the loss of the syndecan-1 encoding sequence.

## Discussion

We report here the results of molecular phylogenetic and comparative genomic analyses of members of the syndecan family of proteoglycans. The presence of syndecans in organisms from Cnidaria to mammals establishes firmly that the syndecan family is ancient in the animal lineage. The Cnidaria, comprising corals, jellyfish, sea anemones and hydroids, are typified by diploblasty, ability for sexual and asexual reproduction and relatively simple body plans. The divergence of the Cnidaria and Bilateria is thought to have taken place between 650–1000 million years ago (MYA) [43]. Nevertheless, genome and EST project have revealed a high level of conservation of Cnidarian genes and their coding sequences with those of vertebrates, including integrins and components of the extracellular matrix [42,64]. The identification of syndecan-encoding sequences in multiple species of Cnidaria provides a strong indication that syndecans, like integrins, have participated as mediators of extracellular interactions throughout animal evolution. The data also establish firmly that the encoding of mutiple syndecans in a single genome is a vertebrate-specific attribute: the genomes of two basal chordates, *Ciona intestinalis *and *Ciona savignyi*, were each found to encode a single syndecan, and single syndecans were identified from the draft genome of the sea urchin *S. purpuratus*, the Cnidarian species, and the amphioxus *B. floridae *EST dataset (Table 1).

The molecular phylogenetic studies demonstrated that syndecan extracellular domains have diverged rapidly in sequence – e.g., each of the invertebrate syndecans has an extracellular domain that is distinct from other species and from all the vertebrate syndecans – whereas the cytoplasmic domains contain highly conserved elements. Identification of syndecans across phyla thus relies heavily on recognition of the cytoplasmic domains in database searches. Within all cytoplasmic domains, the C1 and C2 regions are extremely well-conserved. With regard to the distinctive V regions, our study identified two residues that are extremely highly conserved: the first leucine residue, (corresponding to L289 of mouse syndecan-1) that is universally conserved and the tyrosine residue (corresponding to Y300 of mouse syndecan-1) that is conserved throughout the Bilateria but not in Cnidaria (Figure 1). Current knowledge of vertebrate syndecans suggests that these residues could serve either structural or functional roles. The syndecan-4 cytoplasmic domain forms a homodimer in which L186 is important for the stability of the structure because it makes three contacts with the C1 and V regions of the partner cytoplasmic domain [65]. Although other syndecan cytoplasmic domains are not known to form dimers, it is conceivable that the corresponding leucines could make similar contacts with heterologous binding proteins. Y192 in syndecan-4 also makes three contacts within the homodimer [65]. Thus, in syndecan-4 the most critical roles of L186 and Y192 are structural. In contrast, Y300 in syndecan-1 has been implicated in several signaling roles: it appears necessary for the alignment of syndecan-1 with actin stress fibres [66] and a phosphomimetic Y300E mutant inhibits the assembly of lamellipodia and actin-and-fascin bundles by activated syndecan-1 [13]. It is possible that the presence of this additional tyrosine conferred additional signaling properties on syndecans in the Bilateria. The perspective provided by the new syndecan protein sequence dataset will help guide further experimental analysis of syndecan cytoplasmic domains.

To understand the relationships between the four tetrapod syndecans and gain more insight into the origins of fish syndecans, we turned to a comparative genomic analysis of the vertebrate syndecans. The conservation of synteny is a powerful way to resolve the relationships between paralogous and orthologous members of multigene families in different species [67]. Previous phylogenetic coding sequence analyses have provided indications of paralogy within the family, but the data were not conclusive [68,69]. The loci of the syndecan-1, -2, -3 and -4 genes each showed striking synteny in human, mouse and chicken. With reference to the syndecan genes alone, only partial synteny was detected in fish: whereas in *T. nigroviridis *and *D. rerio *the context of *SDC3 *was clearly syntenic, synteny of *SDC2 *was partial in *T. nigroviridis *andabsent in *D. rerio*. Synteny in fish could not be addressed for the absent syndecan-1 and the unmapped syndecan-4. As discussed below, we achieved resolution of this question by the combined analysis of syndecan and matrilin genes in fish.

**Figure 7:**
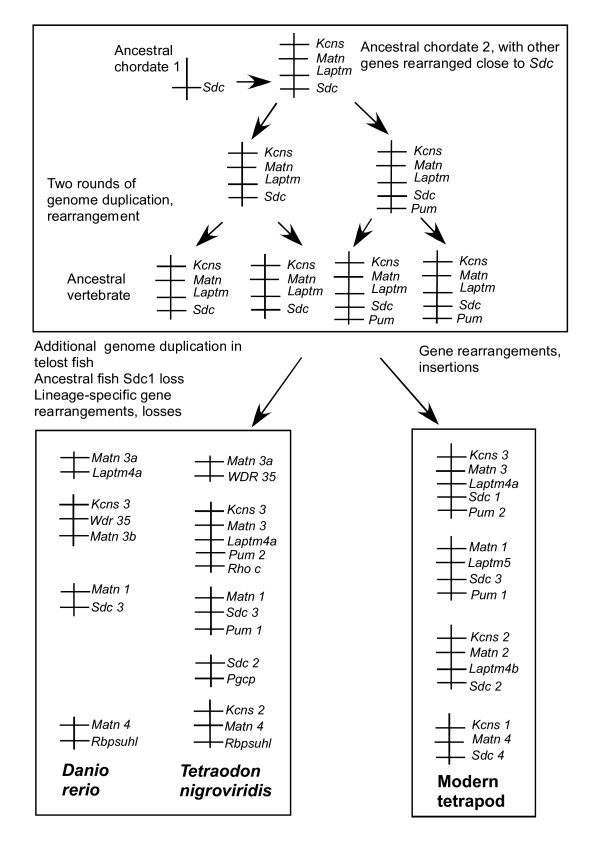
**Model for the evolution of syndecans in fish and tetrapods**. The upper panel of the model shows the hypothesized ancestral genomic context of a single syndecan gene, originating in an ancestral chordate (chordate 2) subsequent to the divergence of the Urochordate lineage. The model assumes that four-fold paralogy was then set up in an ancestral vertebrate as a result of two rounds of whole-genome duplication. This process also sets up pairing within each set of paralogues. The lower right hand panel represents how the initial complete paralogy has degenerated through gene rearrangements in modern tetrapods. Fish underwent an additional round of genome duplication that would have generated additional paralogous pairs [58]. The lower left-hand panel represents the situation in two modern fish, in which only the two matrilin-3 paralogues have been retained from FSGD. The syndecan-1 locus appears to have been lost early in the fish lineage. Other gene rearrangements or losses appear specific to the zebrafish or pufferfish lineages.

The identification of conserved neighboring genes in multiple genomes also revealed a deeper level of homology between all four loci within a single genome, in that most of the conserved neighboring genes also corresponded to paralogous members of gene families. This indication that the four syndecan genes are located in paralogous chromosomal regions was confirmed on the basis of an independent, computationally-based method of analysis of the human genome [57]. When the loci of the four mouse syndecan genes were identified by inter-species backcross mapping, it was noted that the syndecan-1, -2 and -3 genes were syntenic with three members of the myc gene family [70]. There is now strong evidence that paralogous regions exist in vertebrate genomes as a result of whole genome duplications that took place in a vertebrate ancestor after the divergence of the amphioxus lineage [53,57-59]. It appears that the genomic regions of tetrapod syndecan genes have been very well-conserved subsequent to these duplication events. At protein sequence level, there is evidence of pairing within the syndecan family, such that syndecan-1 and -3 are more closely related, as are syndecan-2 and -4 [3,4]. These pairings are also evident at genomic level: the members of a two gene family, *PUM1 *and *PUM2*, are encoded adjacent *SDC3 *and *SDC1*, respectively, and *STK3 *and *STK4 *are encoded adjacent *SDC2 *and *SDC4 *(Figure 4 and Figure 5). Furthermore, the matrilin family also contains pairs: matrilin-1 and -3 are more closely related, as are matrilin-2 and -4 [55,56]. Collectively, these findings are incorporated into a model for the expansion of the syndecan family from a single syndecan in an ancestral chordate to the current multigene status in modern tetrapods and fish (Figure 7).

A surprising and important outcome of the molecular phylogenetic and genomic analyses was the absence of syndecan-1 from the genomes of multiple species of fish (*D. rerio, T. nigroviridis and T. rubripes*). No syndecan-1-like sequences were identified in the three fish genomes, using either amphibian, chicken, or human syndecan-1 as the query sequences. Searches of dbEST, which includes ESTs from additional bony fish species, also did not provide any evidence for a fish syndecan-1. However, all four matrilins are represented in fish (Fig. 6) [62]. Through analysis of the genomic contexts of fish matrilin genes, we were able to strengthen the evidence for conservation of the respective genomic regions between fish and tetrapods. The fish-specific genome duplication (FSGD) that is estimated to have taken place around 320 MYA [63,71] is assumed to have given rise to the paralogous *MATN3a *and *MATN3b *genes. We examined in detail the genomic region around the *MATN3 *genes, as the expected locus of the syndecan-1 gene. The comparison of the loci of *MATN3 *genes in *T. nigroviridis *and *D. rerio *revealed that the genomic region appears to have become split after the genome duplication. An alternative possibility that cannot be excluded on current data is that different sets of genes have been retained alongside *MATN3a *and *MATN3b *in each chromosome after the FSGD. Other gene losses or rearrangements (e.g. loss of *PUM2 *from the *D. rerio *genome, relocation of *WDR35 *to a different chromosome in *T. nigroviridis*; Figure 6) appear lineage-specific and therefore, we infer, were of more recent occurence. From the fossil record, the zebrafish and pufferfish lineages are estimated to have diverged around 284–296 MYA [71]. Thus the loss of *SDC1 *appears, in evolutionary terms, to have occurred relatively soon after FSGD. It is possible that *SDC1 *was lost before, or at the time of, FSGD, however, the available genomes only sample a portion of the fish lineage. Information on the genomes of more basally diverging species of bony fish and a cartilagenous fish would be required to distinguish between these possibilities. Our working model for the current status of fish syndecan genes incorporates the notion of extensive gene loss after FSGD (Figure 7).

Our findings have several practical implications for future studies of syndecan function. While the zebrafish is an excellent vertebrate model organism for most developmental and disease processes [72], it would not be the model of choice for physiological *in vivo *studies of syndecan-1. There is evidence from human, flies and *C. elegans *that adjacent genes can show correlated expression and, in some instances, function in the same pathway [73,74]. The possibility of co-expression or functional association between syndecans and matrilins has not been considered, yet it is intriguing that both are ECM-associated proteins with roles in cell interactions during development and in disease.

## Conclusion

The syndecans are an ancient family of cell adhesion and signaling proteins in animals. The cytoplasmic domains contain very highly conserved sequences and the extracellular domains have undergone rapid change. The four syndecan genes of vertebrates are syntenic across tetrapods, and synteny of the syndecan-2 and -3 genes is also apparent between fish and tetrapods. Each of the four family members are encoded with paralogous genomic regions in which members of the matrilin family are also syntenic between tetrapods and fish. This genomic organization appears to have been set up after the divergence of urochordates (*Ciona*) and vertebrates. The syndecan-1 gene appears to have been lost relatively early in the fish lineage. These conclusions provide the basis for a new model of syndecan evolution in vertebrates and a new perspective for experimental analysis of the roles of syndecans in cells and whole organisms.

## Methods

### Syndecan dataset and molecular phylogeny

#### A. accession numbers

The accession numbers of syndecan and matrilin family members from different organisms, except *T. nigroviridis *and *C. intestinalis*, were obtained by BLASTP and TBLASTN searches of GenBank and dbEST at NCBI. The accession numbers of *T. nigroviridis *syndecans were obtained by BLAST searches of the *T. nigroviridis *genome assembly [75]. *C. intestinalis *syndecan was identified by BLAST searches of the genome and the Gene Clusters [54,76]. The complete datasets are listed in Table 1 and 3. Syndecans from additional species were identified by TBLASTN searches of dbEST: the species included in Table 1 were selected as representatives of additional phyla and sub-phyla for our dataset.

#### B. BLAST searches

The full-length amino acid sequences of syndecan family members from either mouse or human were used to search for syndecans in other species using BLASTP or TBLASTN alignment algorithms at GenBank, their genome databases, and dbEST. The following genome assemblies were searched: *H. sapiens *(NCBI build 35.1) [77,78], *M. musculus *(NCBI build 34 of May 2005) [79], *G. gallus *(NCBI build 1.1, March 2004) [80], *T. rubripes *[81] and *D. melanogaster *(build 4.1, February 2005) [82] at NCBI Blast 2.2.12. *D. rerio *(Zv5 assembly of August 2005) was searched at EBI. The *C. elegans *genome [83] was searched at Sanger worm informatics website (WS149; assembly of Sept, 2005). The *T. nigroviridis *genome [75] (assembly of April, 2004), *X. tropicalis *genome assembly v4.1 [50] and *C. intestinalis *genome and EST database [54,76] were also accessed. Sequences including the cytoplasmic domains of representatives of planarian (*Schmidtea mediterranea*) [84], molluscan (*Euprymna scolopes*), chelicerate (*Rhipicephalus appendiculatus*) [85], and crustacean (*Marsupenaeus japonicus*) syndecans were identified in dbEST and the complete sequence of amphioxus (*Branchiostoma floridae*) syndecan was compiled from over-lapping ESTs in dbEST [[53] and Yu, J., Holland, L.Z., Shin-i, T., Kohara, Y., Satou, Y. and Satoh, N. Expressed genes in *Branchiostoma floridae *project]. Cnidarian syndecan sequences, from *Acropora tenuis *[86], *Acropora palmata *(Schwarz, J.A., Brokstein, P., Manohar, C., Coffroth, M.A., Szmant, A. and Medina, M, Coral-Symbiodinium EST Project), *Hydra magnipapillata *[Bode et al., WashU Hydra EST project], and *Nematostella vectensis *[43], were identified first by TBLASTN search of Stellabase [87] and Cnidbase [88] with known invertebrate syndecans and then confirmed and extended by BLASTN and TBLASTN searches of dbEST.

#### C. multiple alignment

Predicted amino acid sequences of the cytoplasmic domains of syndecans from organisms representing different phyla were aligned in CLUSTALW [46] and are presented in Boxshade 3.2. Predicted amino acid sequences of full-length syndecans were aligned in TCOFFEE (version_2.11) with default parameters using pairwise methods [48].

#### D. tree building

Several parallel methods were used to assess molecular phylogenetic relationship of syndecans, using either the full-length sequences or the cytoplasmic domains. An unrooted phylogenetic tree was constructed from the Phylip distance matrix output of a TCOFFEE alignment [48] in DRAWTREE, and is presented in Phylodendron (D.G. Gilbert, version 0.8d) with the choice of intermediate node positions and node lengths for tree growth [89]. Aligned amino acid sequences of the full-length and cytoplasmic domains of syndecans were analyzed using PHYML, a maximum likelihood reconstruction method, with the WAG substitution model [51]. Bootstrap proportion was used to assess the strength of the topologies for this method [52]. The output data was used to obtain a consensus tree based upon majority extended rule. The consensus outree file (in newick format) was used to prepare an unrooted tree in Phylodendron.

#### E. WU-BLAST

The newly-identified syndecans from representative invertebrates, urochordate, amphibian and fish were compared with the well-characterized human syndecans using WU-Blast 2.0 against the Uniprot dataset [90,91]. The conservation scores were calculated by dividing the bit score of each hit by the maximum bit score value obtained from the sequence used to conduct the WU-BLAST search [92].

## Identification of synteny between syndecan genes

The chromosomal locations of syndecan genes were identified in the physically-mapped genomes of human [93], mouse [94], chicken [95], *T. nigroviridis *[75] and *D. rerio *(EBI assembly Zv5) by TBLASTN searches of each syndecan against the relevant genome. Genes located on either side of the target syndecan gene, (25 on each side, total 50), on the same contig were identified and compared between organisms. In the case of different gene nomenclatures in different organisms, the encoded protein sequences were used in BLASTP or TBLASTN searches against the human genome assembly, to identify the corresponding human gene and its locus. The HUGO gene names are given in the figures.

## Assessment of paralogy

To identify whether the syndecan and matrilin genes are present in paralogous regions of the human genome, the "dataset of paralogons in the human genome, v5.28" was searched [57]. These blocks were defined by McLysaught et al., using a coding sequence cutoff value of e = 10^-7^. Because of the low sequence identity of syndecan extracellular domains, syndecan-1 and -3 were not reproducibly recognized by these criteria. All syndecan genes are included in all blocks in Figure 5.

## Abbreviations

BLAST, **B**asic **L**ocal **A**lignment **S**earch **T**ool; dbEST, **d**ata**b**ase of **E**xpressed **S**equence **T**ags; EBI, **E**uropean **B**ioinformatics **I**nstitute; ECM, **E**xtra**c**ellular **m**atrix; EST, **E**xpressed **S**equence **T**ag; FSGD, **F**ish **S**pecific **G**enome **D**uplication; HUGO, **Hu**man **G**enome **O**rganisation; MYA, **M**illion of **Y**ears **A**go; NCBI, **N**ational **C**enter for **B**iotechnology **I**nformation; PDZ protein, **P**SD-95, **D**isks-large and **Z**O1 protein.

## Authors' contributions

RC contributed data acquisition and analysis, prepared 5 figures and 3 tables, and contributed in the writing of the manuscript. JCA contributed guidance of the project and data analysis, prepared 2 figures and contributed in the writing and final organization of the manuscript.

## Figures and Tables

**Table 1:** Accession numbers of Syndecans.

**Species**	**Syndecan-1**	**Syndecan-2**	**Syndecan-3**	**Syndecan-4**	**References**
*H. sapiens*	NP_002988	NM_002998	NM_14654	NM_002999	96–99
*M. musculus*	NM_011519	NM_011519	NM_011520	NM_011521	100, 101
*G. gallus*	XM_419972	NM_001001462	NM_205383	NM_001007869	101–104
*D. rerio*	Not found	NM_173223	XM_695352	XM_691930	17
*T. nigroviridis*	Not found	GSTENP000-18305001	GSTENT003-1504001	GSTENP0001-1022001	75
*X. laevis*	AAB81324	AAB81325	AAB81326	BU915423, CF285173	49, 105
*X. tropicalis*	Scaffold 100*	NM_001017163	Scaffold 411*	Scaffold 334*	50
*B. floridae*	B1386782, BW711533, BW936816 and others				53
*C. intestinalis*	KYOTOGRAIL2005.125.4.2, Gene collection ID: CiGC35101				54, 76
*C. savignyi*	AB021960				106
*A. crassispina*	AB063328				107
*S. purpuratus*	XM_776754				
*D. melanogaster*	NM_166449				108
*M. japonicus*	CI998451				
*R. appendiculatus*	CD794925, CD788787				85
*E. scolopes*	DW251986				
*C. elegans*	P50605				8, 9, 83
*S. mediterranea*	DN308360				84
*H. magnipapillata*	DT612491				
*N. vectensis*	50645				43
*A. tenuis*	BAE46797				86, 88
*A. palmata*	DR9839838, DR984551				88

All numbers are GenBank Accession numbers, except for *T. nigroviridis *numbers from the Genoscope database and *N. vectensis *from Stellabase. * scaffold numbers refer to *X. tropicalis *genome assembly v4.1 (JGI).

**Table 2:** Sequence conservation of representative invertebrate, fish and amphibian syndecans.

**Organism**	**Human-S1**	**Human-S2**	**Human-S3**	**Human-S4**
*C. savignyi*	0.229	0.201	0.219	0.170
*A. crassispina*	0.190	0.176	-	0.131
*D. melanogaster*	0.204	0.192	0.179	0.175
*C. elegans*	0.182	0.150	-	0.112
*T. nigroviridis, sdc2*	0.230	0.260	0.180	0.190
*T. nigroviridis, sdc3*	0.241	0.301	0.335	0.206
*T. nigroviridis, sdc4*	0.210	0.240	0.187	0.292
*X. laevis, sdc1*	0.363	0.207	0.267	0.170
*X. laevis, sdc2*	0.224	0.485	0.222	0.247
*X. laevis, sdc3*	0.260	0.238	0.554	0.196

Amino acid sequences of human syndecans were used to conduct BlastP searches against the Uniprot database. Conservation scores of relevant retrieved hits (representative of invertebrate, urochordate, fish and amphibian) were then calculated [92]. Dashes indicate searches for which no hit with syndecan-3 was retrieved.

**Table 3:** Accession numbers of Matrilins.

**Species**	**Matrilin-1**	**Matrilin-2**	**Matrilin-3**	**Matrilin-4**	**References**
*H. sapiens*	NM_002379	XM_209824	NM_002381	NM_003833	55–56, 109–11
*M. musculus*	NM_010769	NM_016762	NM_010770	NM_013592	55–56, 110
*G. gallus*	NM_001030375	XM_422012	NM_205072	XM_425698	80, 112
*D. rerio*	BC045465	-	NM_001004007(3a) NM_001012385 (3b)	NM_213549	62
*T. nigroviridis*	GSTENT0003-1505001	GSTENT000-24883001	GSTENT000347-51001 (3a) GSTENT000045-8001	GSTENT000-09286001	75

All numbers are GenBank Accession numbers, except for *T. nigroviridis *numbers from the Genoscope database.

## References

[B1] Fernandez-Busquets X, Burger MM (1999). Cell adhesion and histocompatibility in sponges. Microsc Res Tech.

[B2] Bucior I, Burger MM (2004). Carbohydrate-carbohydrate interaction as a major force initiating cell-cell recognition. Glycoconj J.

[B3] Bernfield M, Gotte M, Park PW, Reizes O, Fitzgerald ML, Lincecum J, Zako M (1999). Functions of cell surface heparan sulfate proteoglycans. Annu Rev Biochem.

[B4] Couchman JR (2003). Syndecans: proteoglycan regulators of cell-surface microdomains?. Nat Rev Mol Cell Biol.

[B5] Steigemann P, Molitor A, Fellert S, Jackle H, Vorbruggen G (2004). Heparan sulfate proteoglycan syndecan promotes axonal and myotube guidance by slit/robo signalling. Curr Biol.

[B6] Fox AN, Zinn K (2005). The heparan sulfate proteoglycan syndecan is an *in vivo *ligand for the *Drosophila *LAR receptor tyrosine phosphatase. Curr Biol.

[B7] Rawson JM, Dimitroff B, Johnson KG, Rawson JM, Ge X, Van Vactor D, Selleck SB (2005). The heparan sulfate proteoglycans Dally-like and Syndecan have distinct functions in axon guidance and visual-system assembly in *Drosophila*. Curr Biol.

[B8] Rhiner C, Gysi S, Frohli E, Hengartner MO, Hajnal A (2005). Syndecan regulates cell migration and axon guidance in *C. elegans*. Development.

[B9] Minniti AN, Labarca M, Hurtado C, Brandan E (2004). *Caenorhabditis elegans *syndecan (SDN-1) is required for normal egg laying and associates with the nervous system and the vulva. J Cell Sci.

[B10] Li Q, Park PW, Wilson CL, Parks WC (2002). Matrilysin shedding of syndecan-1 regulates chemokine mobilization and transepithelial efflux of neutrophils in acute lung injury. Cell.

[B11] Stepp MA, Gibson HE, Gala PH, Iglesia DD, Pajoohesh-Ganji A, Pal-Ghosh S, Brown M, Aquino C, Schwartz AM, Goldberger O, Hinkes MT, Bernfield M (2002). Defects in keratinocyte activation during wound healing in the syndecan-1-deficient mouse. J Cell Sci.

[B12] Beauvais DM, Burbach BJ, Rapraeger AC (2004). The syndecan-1ectodomain regulates alphavbeta3 integrin activity in human mammary carcinoma cells. J Cell Biol.

[B13] Chakravarti R, Sapountzi V, Adams JC (2005). Functional role of syndecan-1 cytoplasmic V region in lamellipodial spreading, actin bundling, and cell migration. Mol Biol Cell.

[B14] Kramer KL, Barnette JE, Yost HJ (2002). PKCgamma regulates syndecan-2 inside-out signalling during xenopus left-right development. Cell.

[B15] Ethell IM, Irie F, Kalo MS, Couchman JR, Pasquale EB, Yamaguchi Y (2001). EphB/syndecan-2 signalling in dendritic spine morphogenesis. Neuron.

[B16] Klass CM, Couchman JR, Woods A (2000). Control of extracellular matrix assembly by syndecan-2 proteoglycan. J Cell Sci.

[B17] Chen E, Hermanson S, Ekker SC (2004). Syndecan-2 is essential for angiogenic sprouting during zebrafish development. Blood.

[B18] Reizes O, Lincecum J, Wang Z, Goldberger O, Huang L, Kaksonen M, Ahima R, Hinkes MT, Barsh GS, Rauvala H, Bernfield M (2001). Transgenic expression of syndecan-1 uncovers a physiological control of feeding behavior by syndecan-3. Cell.

[B19] Strader AD, Reizes O, Woods SC, Benoit SC, Seeley RJ (2004). Mice lacking the syndecan-3 gene are resistant to diet-induced obesity. J Clin Invest.

[B20] Wilcox-Adelman SA, Denhez F, Goetinck PF (2002). Syndecan-4 modulates focal adhesion kinase phosphorylation. J Biol Chem.

[B21] Echtermeyer F, Streit M, Wilcox-Adelman S, Saoncella S, Denhez F, Detmar M, Goetinck P (2001). Delayed wound repair and impaired angiogenesis in mice lacking syndecan-4. J Clin Invest.

[B22] Bobardt MD, Saphire AC, Hung HC, Yu X, Van der Schueren B, Zhang Z, David G, Gallay PA (2003). Syndecan captures, protects, and transmits HIV to T lymphocytes. Immunity.

[B23] Park PW, Pier GB, Hinkes MT, Bernfield M (2001). Exploitation of syndecan-1 shedding by Pseudomonas aeruginosa enhances virulence. Nature.

[B24] Park PW, Foster TJ, Nishi E, Duncan SJ, Klagsbrun M, Chen Y (2004). Activation of syndecan-1 ectodomain shedding by Staphylococcus aureus alpha-toxin and beta-toxin. J Biol Chem.

[B25] Haynes A, Ruda F, Oliver J, Hamood AN, Griswold JA, Park PW, Rumbaugh KP (2005). Syndecan 1 shedding contributes to Pseudomonasaeruginosa sepsis. Infect Immun.

[B26] Alexander CM, Reichsman F, Hinkes MT, Lincecum J, Becker KA, Cumberledge S, Bernfield M (2000). Syndecan-1 is required for Wnt-1-induced mammary tumorigenesis in mice. Nat Genet.

[B27] Dhodapkar MV, Abe E, Theus A, Lacy M, Langford JK, Barlogie B, Sanderson RD (1998). Syndecan-1 is a multifunctional regulator of myeloma pathobiology: control of tumor cell survival, growth, and bone cell differentiation. Blood.

[B28] Joensuu H, Anttonen A, Eriksson M, Makitaro R, Alfthan H, Kinnula V, Leppa S (2002). Soluble syndecan-1 and serum basic fibroblast growth factor are new prognostic factors in lung cancer. Cancer Res.

[B29] Yang Y, Yaccoby S, Liu W, Langford JK, Pumphrey CY, Theus A, Epstein J, Sanderson RD (2002). Soluble syndecan-1 promotes growth of myeloma tumors *in vivo*. Blood.

[B30] Leivonen M, Lundin J, Nordling S, von Boguslawski K, Haglund C (2004). Prognostic value of syndecan-1 expression in breast cancer. Oncology.

[B31] Maeda T, Alexander CM, Friedl A (2004). Induction of syndecan-1 expression in stromal fibroblasts promotes proliferation of human breast cancer cells. Cancer Res.

[B32] Juuti A, Nordling S, Lundin J, Louhimo J, Haglund C (2005). Syndecan-1 expression-a novel prognostic marker in pancreatic cancer. Oncology.

[B33] Lovell R, Dunn JA, Begum G, Barth NJ, Plant T, Moss PA, Drayson MT, Pratt G (2005). Soluble syndecan-1 level at diagnosis is an independent prognostic factor in multiple myeloma and the extent of fall from diagnosis to plateau predicts for overall survival. Br J Haematol.

[B34] Vassilakopoulos TP, Kyrtsonis MC, Papadogiannis A, Nadali G, Angelopoulou MK, Tzenou T, Dimopoulou MN, Siakantaris MP, Kontopidou FN, Kalpadakis C, Kokoris SI, Dimitriadou EM, Tsaftaridis P, Pizzolo G, Pangalis GA (2005). Serum levels of soluble syndecan-1 in Hodgkin's lymphoma. Anticancer Res.

[B35] Gotte M (2003). Syndecans in inflammation. FASEB J.

[B36] Nikkari ST, Jarvelainen HT, Wight TN, Ferguson M, Clowes AW (1994). Smooth muscle cell expression of extracellular matrix genes after arterial injury. Am J Pathol.

[B37] Cizmeci-Smith G, Langan E, Youkey J, Showalter LJ, Carey DJ (1997). Syndecan-4 is a primary-response gene induced by basic fibroblast growth factor and arterial injury in vascular smooth muscle cells. Arterioscler Thromb Vasc Biol.

[B38] Wang H, Moore S, Alavi MZ (1997). Expression of syndecan-1 in rabbit neointima following de-endothelialization by a balloon catheter. Atherosclerosis.

[B39] Filla MS, Dam P, Rapraeger AC (1998). The cell surface proteoglycan syndecan-1 mediates fibroblast growth factor-2 binding and activity. J Cell Physiol.

[B40] McFall AJ, Rapraeger AC (1998). Characterization of the high affinity cell-binding domain in the cell surface proteoglycan syndecan-4. J Biol Chem.

[B41] Zimmermann P, Zhang Z, Degeest G, Mortier E, Leenaerts I, Coomans C, Schulz J, N'Kuli F, Courtoy PJ, David G (2005). Syndecan recycling [corrected] is controlled by syntenin-PIP2 interaction and Arf6. Dev Cell.

[B42] Kortschak RD, Samuel G, Saint R, Miller DJ (2003). EST analysisof the cnidarian *Acropora millepora *reveals extensive gene loss and rapid sequence divergence in the model invertebrates. Curr Biol.

[B43] Darling JA, Reitzel AR, Burton PM, Mazza ME, Ryan JF, Sullivan JC, Finnerty JR (2005). Rising starlet: the starlet sea anemone, *Nematostella vectensis*. Bioessays.

[B44] Koonin EV (2005). Orthologs, paralogs, and evolutionary genomics. Annu Rev Genet.

[B45] Lopez R, Silventoinen V, Robinson R, Kibria A, Gish W (2003). WU-Blast2 server at the European Bioinformatics Institute. Nucleic Acids Research.

[B46] Thompson JD, Higgins DG, Gibson TJ (1994). CLUSTAL W: improving the sensitivity of progressive multiple sequence alignment through sequence weighting, position-specific gap penalties and weight matrix choice. Nucleic Acids Res.

[B47] Harris BZ, Lim WA (2001). Mechanism and role of PDZ domains in signalling complex assembly. J Cell Sci.

[B48] Notredame C, Higgins D, Heringa J (2000). T-Coffee: A novel method for multiple sequence alignments. J Mol Biol.

[B49] Teel AL, Yost HJ (1996). Embryonic expression patterns of *Xenopus *syndecans. Mech Dev.

[B50] Klein SL, Strausberg RL, Wagner L, Pontius J, Clifton SW, Richardson P (2002). Genetic and genomic tools for Xenopus research: The NIH Xenopus initiative. Dev Dyn.

[B51] Guindon S, Gascuel O (2003). A simple, fast and accurate algorithm to estimate large phylogenies by maximum likelihood. Syst Biol.

[B52] Felsenstein J (1985). Confidence limits on phylogenies: An approach using the bootstrap. Evolution.

[B53] Panopoulou G, Hennig S, Groth D, Krause A, Poustka AJ, Herwig R, Vingron M, Lehrach H (2003). New evidence for genome-wide duplications at the origin of vertebrates using an amphioxus gene set and completed animal genomes. Genome Res.

[B54] Satou Y, Kawashima T, Shoguchi E, Nakayama A, Satoh N (2005). An integrated database of the ascidian, Ciona intestinalis: towards functional genomics. Zoolog Sci.

[B55] Deak F, Wagener R, Kiss I, Paulsson M (1999). The matrilins: a novel family of oligomeric extracellular matrix proteins. Matrix Biol.

[B56] Wagener R, Ehlen HW, Ko YP, Kobbe B, Mann HH, Sengle G, Paulsson M (2005). The matrilins--adaptor proteins in the extracellular matrix. FEBS Lett.

[B57] McLysaght A, Hokamp K, Wolfe KH (2002). Extensive genomic duplication during early chordate evolution. Nat Genet.

[B58] Abi-Rached L, Gilles A, Shiina T, Pontarotti P, Inoko H (2002). Evidence of en bloc duplication in vertebrate genomes. Nat Genet.

[B59] Dehal P, Boore JL (2005). Two rounds of whole genome duplication in the ancestral vertebrate. PLoS Biol.

[B60] Vienne A, Rasmussen J, Abi-Rached L, Pontarotti P, Gilles A (2003). Systematic phylogenomic evidence of *en bloc *duplication of the ancestral 8p11.21-8p21.3-like region. Mol Biol Evol.

[B61] Bourque G, Zdobnov EM, Bork P, Pevzner PA, Tesler G (2005). Comparative architectures of mammalian and chicken genomes reveal highly varible rates of genomic rearrangements across different lineages. Genome Res.

[B62] Ko YP, Kobbe B, Paulsson M, Wagener R (2005). Zebrafish (*Danio rerio*) matrilins: shared and divergent characteristics with their mammalian counterparts. Biochem J.

[B63] Meyer A, van der Peer Y (2005). From 2R to 3R: evidence for a fish-specific genome duplication (FSGD). BioEssays.

[B64] Brower DL, Brower SM, Hayward DC, Ball EE (1997). Molecular evolution of integrins: genes encoding integrin beta subunits from a coral and a sponge. Proc Natl Acad Sci USA.

[B65] Shin J, Lee W, Lee D, Koo BK, Han I, Lim Y, Woods A, Couchman JR, Oh ES (2001). Solution structure of the dimeric cytoplasmic domain of syndecan-4. Biochemistry.

[B66] Carey DJ, Bendt KM, Stahl RC (1996). The cytoplasmic domain ofsyndecan-1 is required for cytoskeleton association but not detergent insolubility. Identification of essential cytoplasmic domain residues. J Biol Chem.

[B67] Murphy WJ, Pevzner PA, O'Brien SJ (2004). Mammalian phylogenomics comes of age. Trends Genet.

[B68] Gibson TJ, Spring J (2000). Evidence in favour of ancient octaploidy in the vertebrate genome. Biochem Soc Trans.

[B69] Martin A (2001). Is tetrology true? Lack of support for the "one-to-four" rule?. Mol Biol Evol.

[B70] Spring J, Goldberger OA, Jenkins NA, Gilbert DJ, Copeland NG, Bernfield M (1994). Mapping of the syndecan genes in the mouse: linkage with members of the myc gene family. Genomics.

[B71] Hedges SB, Kumar S (2003). Genomic clocks and evolutionary timescales. Trends Genet.

[B72] Teh C, Parinov S, Korzh V (2005). New ways to admire zebrafish: progress in functional genomics research methodology. Biotechniques.

[B73] Cohen BA, Mitra RD, Hughes JD, Church GM (2000). A computational analysis of whole-genome expression data reveals chromosomal domains of gene expression. Nat Genet.

[B74] Fukuoka Y, Inaoka H, Kohane IS (2004). Inter-species differences of co-expression of neighboring genes in eukaryotic genomes. BMC Genomics.

[B75] Jaillon O, Aury JM, Brunet F, Petit JL, Stange-Thomann N, Mauceli E, Bouneau L, Fischer C, Ozouf-Costaz C, Bernot A, Nicaud S, Jaffe D, Fisher S, Lutfalla G, Dossat C, Segurens B, Dasilva C, Salanoubat M, Levy M, Boudet N, Castellano S, Anthouard V, Jubin C, Castelli V, Katinka M, Vacherie B, Biemont C, Skalli Z, Cattolico L, Poulain J, De Berardinis V, Cruaud C, Duprat S, Brottier P, Coutanceau JP, Gouzy J, Parra G, Lardier G, Chapple C, McKernan KJ, McEwan P, Bosak S, Kellis M, Volff JN, Guigo R, Zody MC, Mesirov J, Lindblad-Toh K, Birren B, Nusbaum C, Kahn D, Robinson-Rechavi M, Laudet V, Schachter V, Quetier F, Saurin W, Scarpelli C, Wincker P, Lander ES, Weissenbach J, Roest Crollius H (2004). Genome duplication in the teleost fish *Tetraodon nigroviridis *reveals the early vertebrate proto-karyotype. Nature.

[B76] Dehal P, Satou Y, Campbell RK, Chapman J, Degnan B, De Tomaso A, Davidson B, Di Gregorio A, Gelpke M, Goodstein DM, Harafuji N, Hastings KE, Ho I, Hotta K, Huang W, Kawashima T, Lemaire P, Martinez D, Meinertzhagen IA, Necula S, Nonaka M, Putnam N, Rash S, Saiga H, Satake M, Terry A, Yamada L, Wang HG, Awazu S, Azumi K, Boore J, Branno M, Chin-Bow S, DeSantis R, Doyle S, Francino P, Keys DN, Haga S, Hayashi H, Hino K, Imai KS, Inaba K, Kano S, Kobayashi K, Kobayashi M, Lee BI, Makabe KW, Manohar C, Matassi G, Medina M, Mochizuki Y, Mount S, Morishita T, Miura S, Nakayama A, Nishizaka S, Nomoto H, Ohta F, Oishi K, Rigoutsos I, Sano M, Sasaki A, Sasakura Y, Shoguchi E, Shin-i T, Spagnuolo A, Stainier D, Suzuki MM, Tassy O, Takatori N, Tokuoka M, Yagi K, Yoshizaki F, Wada S, Zhang C, Hyatt PD, Larimer F, Detter C, Doggett N, Glavina T, Hawkins T, Richardson P, Lucas S, Kohara Y, Levine M, Satoh N, Rokhsar DS (2002). The draft genome of *Ciona intestinalis *insights into chordate and vertebrate origins. Science.

[B77] International Human Genome Sequencing Consortium (2001). Initial sequencing and analysis of the human genome. Nature.

[B78] Venter JC, Adams MD, Myers EW, Li PW, Mural RJ, Sutton GG, Smith HO, Yandell M, Evans CA, Holt RA, Gocayne JD, Amanatides P, Ballew RM, Huson DH, Wortman JR, Zhang Q, Kodira CD, Zheng XH, Chen L, Skupski M, Subramanian G, Thomas PD, Zhang J, Gabor Miklos GL, Nelson C, Broder S, Clark AG, Nadeau J, McKusick VA, Zinder N, Levine AJ, Roberts RJ, Simon M, Slayman C, Hunkapiller M, Bolanos R, Delcher A, Dew I, Fasulo D, Flanigan M, Florea L, Halpern A, Hannenhalli S, Kravitz S, Levy S, Mobarry C, Reinert K, Remington K, Abu-Threideh J, Beasley E, Biddick K, Bonazzi V, Brandon R, Cargill M, Chandramouliswaran I, Charlab R, Chaturvedi K, Deng Z, Di Francesco V, Dunn P, Eilbeck K, Evangelista C, Gabrielian AE, Gan W, Ge W, Gong F, Gu Z, Guan P, Heiman TJ, Higgins ME, Ji RR, Ke Z, Ketchum KA, Lai Z, Lei Y, Li Z, Li J, Liang Y, Lin X, Lu F, Merkulov GV, Milshina N, Moore HM, Naik AK, Narayan VA, Neelam B, Nusskern D, Rusch DB, Salzberg S, Shao W, Shue B, Sun J, Wang Z, Wang A, Wang X, Wang J, Wei M, Wides R, Xiao C, Yan C, Yao A, Ye J, Zhan M, Zhang W, Zhang H, Zhao Q, Zheng L, Zhong F, Zhong W, Zhu S, Zhao S, Gilbert D, Baumhueter S, Spier G, Carter C, Cravchik A, Woodage T, Ali F, An H, Awe A, Baldwin D, Baden H, Barnstead M, Barrow I, Beeson K, Busam D, Carver A, Center A, Cheng ML, Curry L, Danaher S, Davenport L, Desilets R, Dietz S, Dodson K, Doup L, Ferriera S, Garg N, Gluecksmann A, Hart B, Haynes J, Haynes C, Heiner C, Hladun S, Hostin D, Houck J, Howland T, Ibegwam C, Johnson J, Kalush F, Kline L, Koduru S, Love A, Mann F, May D, McCawley S, McIntosh T, McMullen I, Moy M, Moy L, Murphy B, Nelson K, Pfannkoch C, Pratts E, Puri V, Qureshi H, Reardon M, Rodriguez R, Rogers YH, Romblad D, Ruhfel B, Scott R, Sitter C, Smallwood M, Stewart E, Strong R, Suh E, Thomas R, Tint NN, Tse S, Vech C, Wang G, Wetter J, Williams S, Williams M, Windsor S, Winn-Deen E, Wolfe K, Zaveri J, Zaveri K, Abril JF, Guigo R, Campbell MJ, Sjolander KV, Karlak B, Kejariwal A, Mi H, Lazareva B, Hatton T, Narechania A, Diemer K, Muruganujan A, Guo N, Sato S, Bafna V, Istrail S, Lippert R, Schwartz R, Walenz B, Yooseph S, Allen D, Basu A, Baxendale J, Blick L, Caminha M, Carnes-Stine J, Caulk P, Chiang YH, Coyne M, Dahlke C, Mays A, Dombroski M, Donnelly M, Ely D, Esparham S, Fosler C, Gire H, Glanowski S, Glasser K, Glodek A, Gorokhov M, Graham K, Gropman B, Harris M, Heil J, Henderson S, Hoover J, Jennings D, Jordan C, Jordan J, Kasha J, Kagan L, Kraft C, Levitsky A, Lewis M, Liu X, Lopez J, Ma D, Majoros W, McDaniel J, Murphy S, Newman M, Nguyen T, Nguyen N, Nodell M, Pan S, Peck J, Peterson M, Rowe W, Sanders R, Scott J, Simpson M, Smith T, Sprague A, Stockwell T, Turner R, Venter E, Wang M, Wen M, Wu D, Wu M, Xia A, Zandieh A, Zhu X (2001). The sequence of the human genome. Science.

[B79] Waterston RH, Lindblad-Toh K, Birney E, Rogers J, Abril JF, Agarwal P, Agarwala R, Ainscough R, Alexandersson M, An P, Antonarakis SE, Attwood J, Baertsch R, Bailey J, Barlow K, Beck S, Berry E, Birren B, Bloom T, Bork P, Botcherby M, Bray N, Brent MR, Brown DG, Brown SD, Bult C, Burton J, Butler J, Campbell RD, Carninci P, Cawley S, Chiaromonte F, Chinwalla AT, Church DM, Clamp M, Clee C, Collins FS, Cook LL, Copley RR, Coulson A, Couronne O, Cuff J, Curwen V, Cutts T, Daly M, David R, Davies J, Delehaunty KD, Deri J, Dermitzakis ET, Dewey C, Dickens NJ, Diekhans M, Dodge S, Dubchak I, Dunn DM, Eddy SR, Elnitski L, Emes RD, Eswara P, Eyras E, Felsenfeld A, Fewell GA, Flicek P, Foley K, Frankel WN, Fulton LA, Fulton RS, Furey TS, Gage D, Gibbs RA, Glusman G, Gnerre S, Goldman N, Goodstadt L, Grafham D, Graves TA, Green ED, Gregory S, Guigo R, Guyer M, Hardison RC, Haussler D, Hayashizaki Y, Hillier LW, Hinrichs A, Hlavina W, Holzer T, Hsu F, Hua A, Hubbard T, Hunt A, Jackson I, Jaffe DB, Johnson LS, Jones M, Jones TA, Joy A, Kamal M, Karlsson EK, Karolchik D, Kasprzyk A, Kawai J, Keibler E, Kells C, Kent WJ, Kirby A, Kolbe DL, Korf I, Kucherlapati RS, Kulbokas EJ, Kulp D, Landers T, Leger JP, Leonard S, Letunic I, Levine R, Li J, Li M, Lloyd C, Lucas S, Ma B, Maglott DR, Mardis ER, Matthews L, Mauceli E, Mayer JH, McCarthy M, McCombie WR, McLaren S, McLay K, McPherson JD, Meldrim J, Meredith B, Mesirov JP, Miller W, Miner TL, Mongin E, Montgomery KT, Morgan M, Mott R, Mullikin JC, Muzny DM, Nash WE, Nelson JO, Nhan MN, Nicol R, Ning Z, Nusbaum C, O'Connor MJ, Okazaki Y, Oliver K, Overton-Larty E, Pachter L, Parra G, Pepin KH, Peterson J, Pevzner P, Plumb R, Pohl CS, Poliakov A, Ponce TC, Ponting CP, Potter S, Quail M, Reymond A, Roe BA, Roskin KM, Rubin EM, Rust AG, Santos R, Sapojnikov V, Schultz B, Schultz J, Schwartz MS, Schwartz S, Scott C, Seaman S, Searle S, Sharpe T, Sheridan A, Shownkeen R, Sims S, Singer JB, Slater G, Smit A, Smith DR, Spencer B, Stabenau A, Stange-Thomann N, Sugnet C, Suyama M, Tesler G, Thompson J, Torrents D, Trevaskis E, Tromp J, Ucla C, Ureta-Vidal A, Vinson JP, Von Niederhausern AC, Wade CM, Wall M, Weber RJ, Weiss RB, Wendl MC, West AP, Wetterstrand K, Wheeler R, Whelan S, Wierzbowski J, Willey D, Williams S, Wilson RK, Winter E, Worley KC, Wyman D, Yang S, Yang SP, Zdobnov EM, Zody MC, Lander ES, Mouse Genome Sequencing Consortium (2002). Initial sequencing and comparative analysis of the mouse genome. Nature.

[B80] Hillier LW, Miller W, Birney E, Warren W, Hardison RC, Ponting CP, Bork P, Burt DW, Groenen MA, Delany ME, Dodgson JB, Chinwalla AT, Cliften PF, Clifton SW, Delehaunty KD, Fronick C, Fulton RS, Graves TA, Kremitzki C, Layman D, Magrini V, McPherson JD, Miner TL, Minx P, Nash WE, Nhan MN, Nelson JO, Oddy LG, Pohl CS, Randall-Maher J, Smith SM, Wallis JW, Yang SP, Romanov MN, Rondelli CM, Paton B, Smith J, Morrice D, Daniels L, Tempest HG, Robertson L, Masabanda JS, Griffin DK, Vignal A, Fillon V, Jacobbson L, Kerje S, Andersson L, Crooijmans RP, Aerts J, van der Poel JJ, Ellegren H, Caldwell RB, Hubbard SJ, Grafham DV, Kierzek AM, McLaren SR, Overton IM, Arakawa H, Beattie KJ, Bezzubov Y, Boardman PE, Bonfield JK, Croning MD, Davies RM, Francis MD, Humphray SJ, Scott CE, Taylor RG, Tickle C, Brown WR, Rogers J, Buerstedde JM, Wilson SA, Stubbs L, Ovcharenko I, Gordon L, Lucas S, Miller MM, Inoko H, Shiina T, Kaufman J, Salomonsen J, Skjoedt K, Wong GK, Wang J, Liu B, Wang J, Yu J, Yang H, Nefedov M, Koriabine M, Dejong PJ, Goodstadt L, Webber C, Dickens NJ, Letunic I, Suyama M, Torrents D, von Mering C, Zdobnov EM, Makova K, Nekrutenko A, Elnitski L, Eswara P, King DC, Yang S, Tyekucheva S, Radakrishnan A, Harris RS, Chiaromonte F, Taylor J, He J, Rijnkels M, Griffiths-Jones S, Ureta-Vidal A, Hoffman MM, Severin J, Searle SM, Law AS, Speed D, Waddington D, Cheng Z, Tuzun E, Eichler E, Bao Z, Flicek P, Shteynberg DD, Brent MR, Bye JM, Huckle EJ, Chatterji S, Dewey C, Pachter L, Kouranov A, Mourelatos Z, Hatzigeorgiou AG, Paterson AH, Ivarie R, Brandstrom M, Axelsson E, Backstrom N, Berlin S, Webster MT, Pourquie O, Reymond A, Ucla C, Antonarakis SE, Long M, Emerson JJ, Betran E, Dupanloup I, Kaessmann H, Hinrichs AS, Bejerano G, Furey TS, Harte RA, Raney B, Siepel A, Kent WJ, Haussler D, Eyras E, Castelo R, Abril JF, Castellano S, Camara F, Parra G, Guigo R, Bourque G, Tesler G, Pevzner PA, Smit A, Fulton LA, Mardis ER, Wilson RK, InternationalChicken Genome Sequencing Consortium (2004). Sequence and comparative analysis of the chicken genome provide unique perspectives on vertebrate evolution. Nature.

[B81] Aparicio S, Chapman J, Stupka E, Putnam N, Chia JM, Dehal P, Christoffels A, Rash S, Hoon S, Smit A, Gelpke MD, Roach J, Oh T, Ho IY, Wong M, Detter C, Verhoef F, Predki P, Tay A, Lucas S, Richardson P, Smith SF, Clark MS, Edwards YJ, Doggett N, Zharkikh A, Tavtigian SV, Pruss D, Barnstead M, Evans C, Baden H, Powell J, Glusman G, Rowen L, Hood L, Tan YH, Elgar G, Hawkins T, Venkatesh B, Rokhsar D, Brenner S (2002). Whole-genome shotgun assembly and analysis of the genome of *Fugu rubripes*. Science.

[B82] Adams MD, Celniker SE, Holt RA, Evans CA, Gocayne JD, Amanatides PG, Scherer SE, Li PW, Hoskins RA, Galle RF, George RA, Lewis SE, Richards S, Ashburner M, Henderson SN, Sutton GG, Wortman JR, Yandell MD, Zhang Q, Chen LX, Brandon RC, Rogers YH, Blazej RG, Champe M, Pfeiffer BD, Wan KH, Doyle C, Baxter EG, Helt G, Nelson CR, Gabor GL, Abril JF, Agbayani A, An HJ, Andrews-Pfannkoch C, Baldwin D, Ballew RM, Basu A, Baxendale J, Bayraktaroglu L, Beasley EM, Beeson KY, Benos PV, Berman BP, Bhandari D, Bolshakov S, Borkova D, Botchan MR, Bouck J, Brokstein P, Brottier P, Burtis KC, Busam DA, Butler H, Cadieu E, Center A, Chandra I, Cherry JM, Cawley S, Dahlke C, Davenport LB, Davies P, de Pablos B, Delcher A, Deng Z, Mays AD, Dew I, Dietz SM, Dodson K, Doup LE, Downes M, Dugan-Rocha S, Dunkov BC, Dunn P, Durbin KJ, Evangelista CC, Ferraz C, Ferriera S, Fleischmann W, Fosler C, Gabrielian AE, Garg NS, Gelbart WM, Glasser K, Glodek A, Gong F, Gorrell JH, Gu Z, Guan P, Harris M, Harris NL, Harvey D, Heiman TJ, Hernandez JR, Houck J, Hostin D, Houston KA, Howland TJ, Wei MH, Ibegwam C, Jalali M, Kalush F, Karpen GH, Ke Z, Kennison JA, Ketchum KA, Kimmel BE, Kodira CD, Kraft C, Kravitz S, Kulp D, Lai Z, Lasko P, Lei Y, Levitsky AA, Li J, Li Z, Liang Y, Lin X, Liu X, Mattei B, McIntosh TC, McLeod MP, McPherson D, Merkulov G, Milshina NV, Mobarry C, Morris J, Moshrefi A, Mount SM, Moy M, Murphy B, Murphy L, Muzny DM, Nelson DL, Nelson DR, Nelson KA, Nixon K, Nusskern DR, Pacleb JM, Palazzolo M, Pittman GS, Pan S, Pollard J, Puri V, Reese MG, Reinert K, Remington K, Saunders RD, Scheeler F, Shen H, Shue BC, Siden-Kiamos I, Simpson M, Skupski MP, Smith T, Spier E, Spradling AC, Stapleton M, Strong R, Sun E, Svirskas R, Tector C, Turner R, Venter E, Wang AH, Wang X, Wang ZY, Wassarman DA, Weinstock GM, Weissenbach J, Williams SM, Woodage T, Worley KC, Wu D, Yang S, Yao QA, Ye J, Yeh RF, Zaveri JS, Zhan M, Zhang G, Zhao Q, Zheng L, Zheng XH, Zhong FN, Zhong W, Zhou X, Zhu S, Zhu X, Smith HO, Gibbs RA, Myers EW, Rubin GM, Venter JC (2000). The genome sequence of *Drosophila melanogaster*. Science.

[B83] *C. elegans* (1998). Sequencing Consortium: Genomesequence of the nematode *C. elegans *a platform for investigating biology. Science.

[B84] Zayas RM, Hernandez A, Habermann B, Wang Y, Stary JM, Newmark PA (2005). The planarian *Schmidtea mediterranea *as a model for epigenetic germ cell specification: Analysis of ESTs from the hermaphroditic strain. Proc Natl Acad Sci USA.

[B85] Nene V, Lee D, Kang'a S, Skilton R, Shah T, de Villiers E, Mwaura S, Taylor D, Quackenbush J, Bishop R (2004). Genes transcribed in the salivary glands of female *Rhipicephalus appendiculatus *ticks infected with *Theileria parva*. Insect Biochem Mol Biol.

[B86] Yuyama I, Hayakawa H, Endo H, Iwao K, Takeyama H, Maruyama T, Watanabe T (2005). Identification of symbiotically expressed coral mRNAs using a model infection system. Biochem Biophys Res Commun.

[B87] Sullivan JC, Ryan JF, Watson JA, Webb J, Mullikin JC, Rokhsar D, Finnerty JR (2006). StellaBase: the *Nematostella vectensis *Genomics Database. Nucleic Acids Res.

[B88] Ryan JF, Finnerty JR (2003). CnidBase: The Cnidarian Evolutionary Genomics Database. Nucleic Acids Res.

[B89] Phylodendron- Phylogenetic tree printer. http://iubio.bio.indiana.edu/treeapp/treeprint-form.html.

[B90] Pillai S, Silventoinen V, Kallio K, Senger M, Sobhany S, Tate J, Velankar S, Golovin A, Henrick K, Rice P, Stoehr P, Lopez R (2005). SOAP-based services provided by the European Bioinformatics Institute. Nucleic Acids Res.

[B91] Lopez R, Silventoinen V, Robinson S, Kibria A, Gish W (2003). WU-Blast2 server at the European Bioinformatics Institute. Nucleic Acids Res.

[B92] Furney SJ, Higgins DG, Ouzounis CA, Lopez-Bigas N (2006). Structural and functional properties of genes involved in human cancer. BMC Genomics.

[B93] McPherson JD, Marra L, Hillier L, Waterston RH, Chinwalla A, Wallis J, Sekhon M, Wylie K, Mardis ER, Wilson RK (2001). A physical map of the human genome. Nature.

[B94] Gregory SG, Sekhon M, Schein J, Zhao S, Osoegawa K, Scott CE, Evans RS, Burridge PW, Cox TV, Fox CA, Hutton RD, Mullenger IR, Phillips KJ, Smith J, Stalker J, Threadgold GJ, Birney E, Wylie K, Chinwalla A, Wallis J, Hillier L, Carter J, Gaige T, Jaeger S, Kremitzki C, Layman D, Maas J, McGrane R, Mead K, Walker R, Jones S, Smith M, Asano J, Bosdet I, Chan S, Chittaranjan S, Chiu R, Fjell C, Fuhrmann D, Girn N, Gray C, Guin R, Hsiao L, Krzywinski M, Kutsche R, Lee SS, Mathewson C, McLeavy C, Messervier S, Ness S, Pandoh P, Prabhu AL, Saeedi P, Smailus D, Spence L, Stott J, Taylor S, Terpstra W, Tsai M, Vardy J, Wye N, Yang G, Shatsman S, Ayodeji B, Geer K, Tsegaye G, Shvartsbeyn A, Gebregeorgis E, Krol M, Russell D, Overton L, Malek JA, Holmes M, Heaney M, Shetty J, Feldblyum T, Nierman WC, Catanese JJ, Hubbard T, Waterston RH, Rogers J, de Jong PJ, Fraser CM, Marra M, McPherson JD, Bentley DR (2002). A physical map of the mouse genome. Nature.

[B95] Wallis JW, Aerts J, Groenen MA, Crooijmans RP, Layman D, Graves TA, Scheer DE, Kremitzki C, Fedele MJ, Mudd NK, Cardenas M, Higginbotham J, Carter J, McGrane R, Gaige T, Mead K, Walker J, Albracht D, Davito J, Yang SP, Leong S, Chinwalla A, Sekhon M, Wylie K, Dodgson J, Romanov MN, Cheng H, de Jong PJ, Osoegawa K, Nefedov M, Zhang H, McPherson JD, Krzywinski M, Schein J, Hillier L, Mardis ER, Wilson RK, Warren WC (2004). A physical map of the chicken genome. Nature.

[B96] Mali M, Jaakkola P, Arvilommi AM, Jalkanen M (1990). Sequence of human syndecan indicates a novel gene family of integral membrane proteoglycans. J Biol Chem.

[B97] David G, van der Schueren B, Marynen P, Cassiman JJ, van den Berghe H (1992). Molecular cloning of amphiglycan, a novel integral membrane heparan sulfate proteoglycan expressed by epithelial and fibroblastic cells. J Cell Biol.

[B98] Kojima T, Inazawa J, Takamatsu J, Rosenberg RD, Saito H (1993). Human ryudocan core protein: molecular cloning and characterization of the cDNA, and chromosomal localization of the gene. Biochem Biophys Res Commun.

[B99] Berndt C, Casaroli-Marano RP, Vilaro S, Reina M (2001). Cloning and characterization of human syndecan-3. J Cell Biochem.

[B100] Saunders S, Jalkanen M, O'Farrell S, Bernfield M (1989). Molecular cloning of syndecan, an integral membrane proteoglycan. J Cell Biol.

[B101] Tsuzuki S, Kojima T, Katsumi A, Yamazaki T, Sugiura I, Saito H (1997). Molecular cloning, genomic organization, promoter activity, and tissue-specific expression of the mouse ryudocan gene. J Biochem.

[B102] Chen L, Couchman JR, Smith J, Woods A (2002). Molecular characterization of chicken syndecan-2 proteoglycan. Biochem J.

[B103] Gould SE, Upholt WB, Kosher RA (1995). Characterization of chicken syndecan-3 as a heparan sulfate proteoglycan and its expression during embryogenesis. Dev Biol.

[B104] Baciu PC, Acaster C, Goetinck PF (1994). Molecular cloning and genomic organization of chicken syndecan-4. J Biol Chem.

[B105] Rosenblum ND, Botelho BB, Bernfield M (1995). Expression of a *Xenopus *counterpart of mammalian syndecan 2 during embryogenesis. Biochem J.

[B106] Satou Y, Chiba S, Satoh N (1999). Expression cloning of an ascidian syndecan suggests its role in embryonic cell adhesion and morphogenesis. Dev Biol.

[B107] Tomita K, Yamasu K, Suyemitsu T (2000). Cloning and characterization of cDNA for syndecan core protein in sea urchin embryos. Dev Growth Differ.

[B108] Spring J, Paine-Saunders SE, Hynes RO, Bernfield M (1994). *Drosophila *syndecan: conservation of a cell-surface heparan sulfate proteoglycan. Proc Natl Acad Sci USA.

[B109] Muratoglu S, Krysan K, Balazs M, Sheng H, Zakany R, Modis L, Kiss I, Deak F (2000). Primary structure of human matrilin-2, chromosome location of the MATN2 gene and conservation of an AT-AC intron in matrilin genes. Cytogenet Cell Genet.

[B110] Mates L, Korpos E, Deak F, Liu Z, Beier DR, Aszodi A, Kiss I (2002). Comparative analysis of the mouse and human genes (Matn2 and MATN2) for matrilin-2, a filament-forming protein widely distributed in extracellular matrices. Matrix Biol.

[B111] Belluoccio D, Schenker T, Baici A, Trueb B (1998). Characterization of human matrilin-3 (MATN3). Genomics.

[B112] Belluoccio D, Trueb B (1997). Matrilin-3 from chicken cartilage. FEBS Lett.

